# Case Report: Disseminated, rifampicin resistant *Mycobacterium bovis* (BCG) infection in an immunocompromised child

**DOI:** 10.12688/wellcomeopenres.16280.1

**Published:** 2020-10-15

**Authors:** Simon B. Drysdale, Dominic F. Kelly, Marcus Morgan, Tim Peto, Derrick Crook, Philippa C. Matthews, Timothy M. Walker

**Affiliations:** 1 Oxford Vaccine Group, Department of Paediatrics, University of Oxford, Oxford, UK; 2 NIHR Oxford Biomedical Research Centre, Oxford University, Oxford, UK; 3 Paediatric Infectious Diseases Research Group, St George's, University of London, London, UK; 4 Department of Microbiology and Infectious Diseases, Oxford University Hospitals NHS Foundation Trust, Oxford, UK; 5 Nuffield Department of Medicine, University of Oxford, Oxford, UK; 6 Oxford University Clinical Research Unit, University of Oxford, Ho Chi Minh City, Vietnam

**Keywords:** BCG, drug resistance, whole genome sequencing, immunocompromised

## Abstract

**Background:** *Bacillus Calmette–Guérin* (BCG) is a live-attenuated vaccine used world-wide for prevention of tuberculosis disease. In some immunocompromised hosts it has the potential to cause disease. As with other members of the *M. tuberculosis *complex it has the potential for acquiring drug resistance.

**Methods: **We reviewed 10 years of paediatric clinical BCG strains referred to our clinical microbiology laboratory in Oxford where they underwent whole genome sequencing. We present a case series comparing clinical, pathogen genetic and pathogen phenotypic data, and consider the clinical implications.

**Results: **We identified 15 BCG isolates from 8 children under 16 years old. Only one child had clinical disease with the other seven reported as local inoculation-site reactions. Case 1 suffered disseminated disease secondary to an undiagnosed IL-12/IFNγ receptor defect and the BCG isolates evolved two different rifampicin resistance mutations. Across all 15 isolates, phenotypic resistance to each first line drug was seen.

**Conclusions: **BCG is a safe and effective vaccine in children. Most clinical specimens in our series were not related to disease. However, in the context of rare pathogen-specific immunocompromise, BCG can cause pathology and acquire drug resistance under selection from therapy.

## Introduction

The *Bacillus Calmette–Guérin* (BCG) vaccine has been in use worldwide for many years as prophylaxis against *Mycobacterium tuberculosis* infection. It is a live-attenuated vaccine derived from many cell culture passages of *Mycobacterium bovis*. There are multiple different *M. bovis* BCG strains ^1^ but the Danish Staten Serum Institute (SSI) 1331 strain has been the most widely used in the United Kingdom (UK).

All *M. bovis* BCG strains are inherently resistant to pyrazinamide. The *in vitro* susceptibility of vaccine strains to other antibiotic agents has been investigated by previous studies ^1, 2^. Unusually, the Danish BCG strain 1331 has only intermediate isoniazid susceptibility (sensitive at 0.4 μg/ml but resistant at 0.1 μg/ml) ^1^ and is resistant to ethionamide. *In vivo*, ** however, serum concentrations of isoniazid would often be >0.1 μg/ml ^3^ and its low-grade resistance has not been considered a clinical problem. Alternative vaccine strains have occasionally been used during periods of interruption to the supply of the Danish 1331 preparation in the UK. These include the InterVax (Canada) BCG vaccine (Bulgarian substrain [Sofia] SL222) ^4^ and the Connaught strain ^2^.

BCG adenitis following vaccination is relatively common, with an incidence of 1 in 1500–2800 ^5^. The history has been well described, and local infection usually regresses spontaneously ^6^. In contrast, disseminated BCG infection is extremely rare (estimated to affect 3.4 children per million given BCG ^5^) and is characteristically associated with primary or secondary immunodeficiency. There are no clear guidelines on how best to treat children with disseminated *M. bovis* BCG strain infection. However, treatment regimens generally include a prolonged course of rifampicin, isoniazid and ethambutol with adjustments made according to antibiotic susceptibility testing, led by an expert in mycobacterial infection, similar to recommendations for treating patients with multidrug-resistant (MDR) *M. tuberculosis* ^7^.

In Oxfordshire, United Kingdom (UK), the annual incidence of TB is relatively low (approximately 4 per 100,000) and children are only given the BCG vaccine if they are in a high-risk group as defined by Public Health England (PHE) guidance ^8^. This study was triggered by the isolation of a rifampicin-resistant BCG strain from a child in Oxfordshire who was vaccinated due to being in a high-risk epidemiological group, and went on to develop disseminated BCG disease. To better contextualise this case, we sought to assess the antibiotic susceptibilities of other *M. bovis* BCG isolates from children submitted to our clinical microbiology laboratory.

## Methods

### Study population

This study was conducted at Oxford University Hospitals NHS Foundation Trust, UK, a large tertiary referral teaching hospital for which specialist services serve a population of approximately 2.5 million people. Clinical isolates referred to the regional laboratory at Oxford University Hospital NHS Foundation Trust, UK, and identified as *Mycobacterium tuberculosis* complex have been whole-genome-sequenced using Illumina technology since 2007 ^9^. Following the identification of an index case of a child with disseminated BCG infection (‘Case one’), we used this sequence database to identify BCG isolates from children, and reviewed clinical notes and routine microbiology results of these cases.

### Phenotypic susceptibility testing

Culture-based susceptibility testing was performed using liquid culture (Bactec MGIT 960) at the Public Health England National Mycobacterial Reference Laboratory, London.

### Mycobacterial sequencing

For isolates identified as BCG, Illumina reads were mapped to a *Mycobacterium bovis* reference genome ( AF2122/97 NC_002945.3) using Stampy (version 1.0.17), with repetitive regions masked. Variant calls were made using SAMtools mpileup (version 0.1.18), based on a minimum depth of 5X and at least one read on each strand. A mixed-call was assigned where the minority allele composed >10% of read depth ^10^. Genotypic susceptibility predictions were based on a catalogue of resistance mutations previously described ^11^.

### Ethics approval

Our retrospective review of laboratory data did not require formal ethical approval but the Caldicott guardian for Oxford University Hospitals NHS Foundation Trust approved the study and we obtained written informed consent from the parents of our index case (‘Case one’). Mycobacterial sequencing was undertaken as part of service development within clinical microbiology.

## Case report and results

### Clinical context of *M. bovis* samples

We identified 15 clinical samples from eight children <16 years old ( Table 1). Case one was a girl of Pakistani descent who first presented at 4 months of age. She had been vaccinated routinely with BCG (Danish strain 1331) soon after birth as she was from a high-risk epidemiological group. She developed disseminated BCG infection and was subsequently diagnosed with an Interleukin-12 receptor B1 (IL-12 RB1) gene mutation, which is known to increase susceptibility to mycobacterial disease ^12^. The samples from the remaining seven cases all arose from either injection site collections or local adenitis, and all arose in children who had received the same vaccine strain.

**Table 1.** Summary of 12 *M. bovis* BCG strain isolates from 8 children in a UK cohort. All children in this series had been immunised with the Danish 1331 *M.bovis* BCG strain.

					Phenotypic drug sensitivity profile			
	Site of BCG detection	Clinical diagnosis	Underlying condition	Outcome	INH	RIF	EMB	PZA
Case 1	Left axillary LN 2013	BCG adenitis	IL12Rb1 defect	Ongoing infection	S	S	R	R
	Left axillary LN 2013	BCG adenitis						
	Gastric aspirate 2014	Disseminated BCG			S	S	R	R
	Gastric aspirate 2014	Disseminated BCG						
	Right axillary LN 2014	Disseminated BCG			S	S	S	R
	Cervical LN 2015	Relapse disseminated BCG			S	R	S	R
	Pus, abdominal wall 2017	Relapse disseminated BCG			S	S	S	R
Case 2	Left axillary LN 2013	BCG adenitis	Metabolic disorder	Resolved infection	S	S	S	R
Case 3	Tissue L arm 2013	BCG adenitis	Nil	Resolved infection	S	S	R	R
	Axillary swab 2013	BCG adenitis	Nil	Resolved infection				
Case 4	Left axillary LN 2013	BCG adenitis	Nil	Resolved infection	S	S	S	R
Case 5	BCG site swab 2015	BCG site abscess	Nil	Resolved infection	R	S	S	R
Case 6	Left axillary LN 2007	BCG adenitis	HIV	Resolved infection	S	S	S	R
Case 7	Gastric aspirate/PEG swab 2009	Abscess over BCG scar	Nil	Resolved infection	S	S	S	R
Case 8	BCG scar 2007	ND	ND	ND	S	S	S	R

Key: LN, lymph node; RIF, Rifampicin; INH, isoniazid; PZA, pyrazinamide; EMB, ethambutol; ND, No data available. Blank indicates not tested.

### Antibiotic management of *M. bovis* in an immuncompromised host

Case one initially received rifampicin (15 mg/kg daily), isoniazid (10 mg/kg daily), pyrazinamide (35 mg/kg daily) and ethambutol (20 mg/kg daily) for presumed *M. tuberculosis* infection. Following identification of the isolate as *M. bovis* by line-probe assay in the National Mycobacterial Reference Laboratory, London, approximately two months into therapy, pyrazinamide was stopped. A month later, phenotypic susceptibilities became available, suggesting resistance to ethambutol. This agent was therefore switched to moxifloxacin (10 mg/kg daily). She received 15 months of therapy and then commenced azithromycin (10 mg/kg daily, three days a week) for prophylaxis against non-tuberculous mycobacterial infection. She subsequently relapsed with disseminated BCG infection nine months after stopping initial antimycobacterial treatment, requiring multiple surgical procedures (drainage of abdominal collections) and prolonged antibiotic therapy, which are still ongoing three years later.

### Antibiotic susceptibility of *M. bovis* isolates in Case one

Phenotypic susceptibility profiles are summarised in Table 1. All 5 of 7 isolates with phenotypic results from this child were reported as isoniazid susceptible. In total, two of five isolates were reported as phenotypically resistant to ethambutol. In the penultimate isolate (2015), *in vitro* rifampicin resistance was also reported. This corresponded to the identification of a rifampicin resistance mutation ( *rpoB* D435V) in the 2015 isolate, a well characterised mutation in the rifampicin resistance determining region that is the target of the widely-used MTB/RIF Xpert assay ^13^. Interestingly, after rifampicin therapy was stopped and the patient suffered a further relapse in 2017, the organism was reported to be phenotypically susceptible and the D435V mutation was no longer present, although an alternative *rpoB* mutation (L430P) was present on WGS, and was also detected by Xpert MTB/RIF at the reference laboratory. The fact that the subsequent isolate had a different mutation is suggestive of a significant ongoing within-host selective pressure. That the rifampicin phenotype was susceptible despite the L430P resistance mutation is not unprecedented ^13^.

### Antibiotic susceptibility of other *M. bovis* isolates

**Figure 1.**
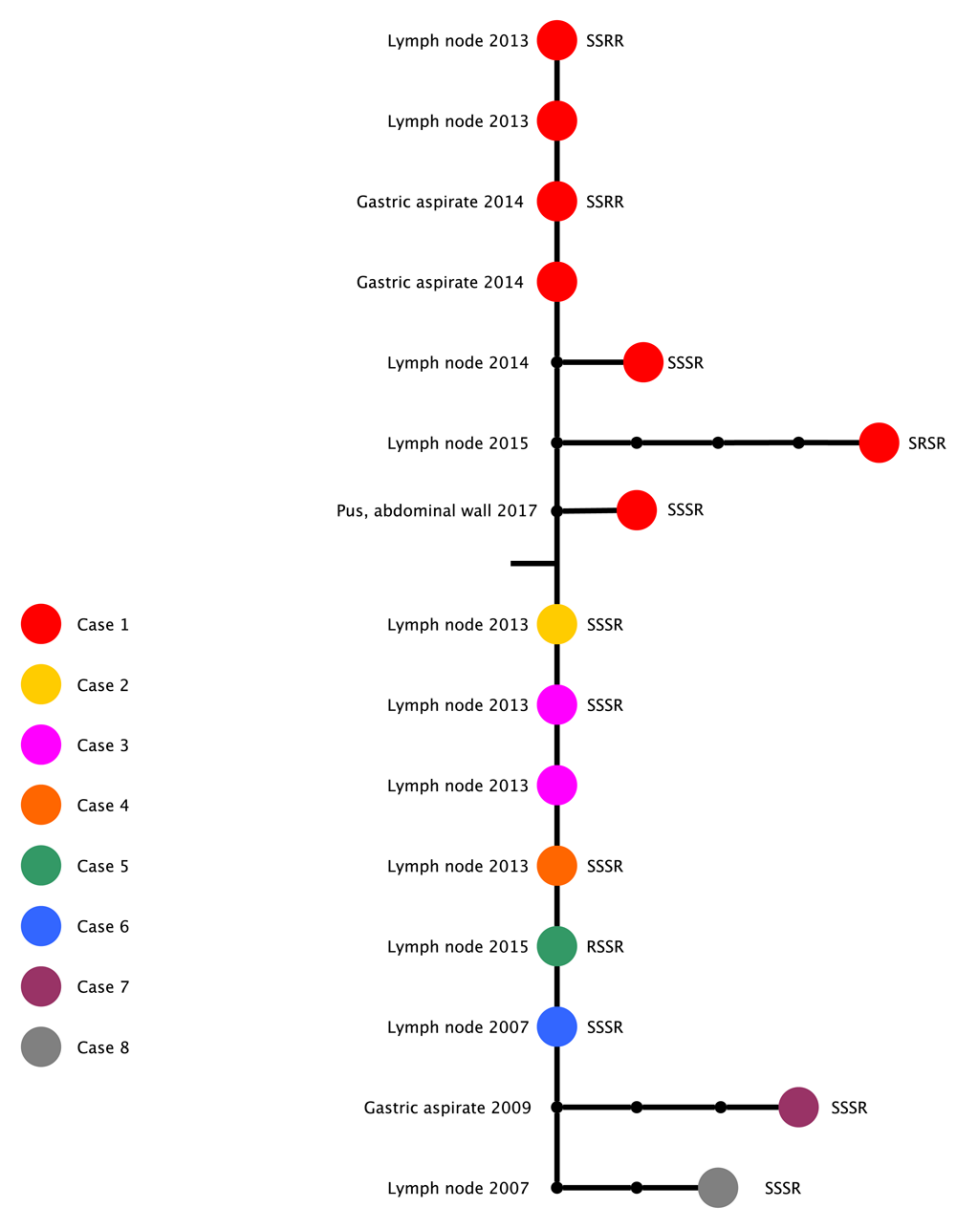
Maximum likelihood phylogenetic tree based on whole genome sequences of BCG isolates. Tip labels describe the case number, site of isolation, susceptibility profile ordered as isoniazid, rifampicin, ethambutol, pyrazinamide, and year of isolation. Scale bar shows a branch length of 0.5 SNPs.

Among the other seven children from whom BCG was isolated, one exhibited phenotypic resistance to ethambutol and one to isoniazid ( Table 1). Despite resistance being reported to all first-line antibiotics within our set of isolates, no single isolate was resistant to more than two drugs (i.e. more than one in addition to pyrazinamide). We were unable to identify any genetic basis for phenotypic resistance to isoniazid or ethambutol, despite searching through a list of known resistance-mechanisms with 94–98% sensitivity in *M. tuberculosis* infection ^13^. As expected, based on derivation from a single vaccine strain, the paediatric isolates were all closely related, with a maximum of seven SNPs separating any two samples ( Figure 1). Interestingly, we observed phenotypic variation in drug susceptibility even among isolates that are genomically indistinguishable.

### Sequence data

All raw sequence data have been uploaded to the ENA and are available under the project number PRJNA548242.

## Discussion

We have confirmed that isolation of *M. bovis* BCG strain from clinical samples is rare, with only eight positive cases recorded over an 11-year period in a large teaching hospital laboratory. The majority of these reflected local adenitis, which is a well-recognised complication of vaccination ^14, 15^. There was only one case of disseminated *M. bovis* BCG, which occurred in a child with an immunodeficiency known to increase susceptibility to severe mycobacterial infection ^12^.

This epidemiology reflects that which has been previously described; in a previous study in London 98% of BCG complications were local, with only 2% having systemic sequelae ^14^. Likewise, National UK Medicines and Healthcare Products Regulatory Agency (MHRA) data from 2013/14 reported 41 adverse incidents related to the BCG vaccine, of which only two were disseminated BCG infection, the rest being local complications ^16^. The Australian mycobacterial reference laboratory detected 24 *M. bovis* BCG isolates in 2009 (nine in children under six years old), with the majority of these being representing vaccine site abscesses (n=13) ^17^. Similarly, in the USA between 2006–2013 there were only 73 cases of *M. bovis* BCG strain isolated compared with 91,985 cases of *M. tuberculosis* ^18^. 

In this study, we are able to draw on full-length sequence data for *M. bovis* to assess the presence of drug-resistance mutations and correlate these with phenotypic resistance testing. Rifampicin resistance in *M. bovis* (BCG) has been reported previously, in each case in an immunocompromised child, including its emergence on treatment ^19– 21^. Case one in our series had multiple isolates tested, with genotypic rifampicin resistance twice selected for, but only detected phenotypically once. This may be due to variations in the subpopulations of *M. bovis* organisms that are sampled in a patient with a high bacterial burden, or reflect antibiotic pressure (the rifampicin our patient had been receiving was stopped when the resistant isolate was detected). The presence of phenotypic resistance to isoniazid and ethambutol despite the absence of any identifiable genetic predictors of resistance is interesting: it might represent hitherto unrecognised mechanisms of resistance, or alternatively error in phenotypic determination of resistance. The latter is suggested by the fact that *in vitro* susceptibility reporting varies despite indistinguishable genomic sequences.

In our small series of cases we have observed *in vitro* resistance to each first-line anti-tuberculosis drug, despite BCG being a laboratory strain that is administered to patients. Despite known low-level isoniazid resistance in the Danish SSI 1331 BCG strain ^19^, to our knowledge no underlying mechanism for this has been identified. Whatever the explanation, as far as we know the presence of resistance to any drug has only been clinically relevant in Case one in our cohort, whose primary problem was one of immunodeficiency predisposing to severe mycobacterial infection. Since local infection commonly either resolves spontaneously, or can be managed by aspiration or surgical excision ^14, 15^, the antibiotic resistance profile may not be clinically relevant to developing a safe management plan.

Although the BCG vaccine is contraindicated in patients with immunodeficiencies, in the UK infants in high-risk groups for developing tuberculosis are usually vaccinated soon after birth, before any immunodeficiency will have become clinically apparent. In the cases with local vaccine site collections or adenitis, it could be argued that laboratory diagnosis did not inform the management, and thus in only one of the seven cases with clinical information available was the mycobacterial culture and/or sequence data clinically useful. 

It is not known how many doses of BCG are given to UK children per year, but according to Public Health England (PHE) data for high-risk areas, it will be considerably more than 30,000 doses ^22^. A recent systematic review and meta-analysis ^23^ of the effect of BCG vaccination in children demonstrated a 19% protective efficacy against tuberculosis infection among vaccinated children after exposure compared with unvaccinated children, and protection by BCG against progression from infection to active disease of 58%. Thus, that only one case had invasive disease from all the children receiving the BCG in our large population catchment area, suggests BCG is safe, and that it is the host response that is important in the development of disease rather than any specific attribute of the vaccine strain. 

In view of the dramatic changes in the development of whole genome sequencing of mycobacteria in the last few years, routine phenotypic antibiotic resistance testing has been discontinued in England in favour of genotypic testing. On the one hand this will provide more reproducible results, but on the other hand it will not detect resistance due to unknown mechanisms, such as that reported for isoniazid in the Danish BCG strain. However, work to enrich our knowledgebase of molecular resistance determinants is accelerating ^13^ and the clinical relevance of this low-level isoniazid resistance that is only rarely detected even by phenotypic methods is questionable. Phenotyping only detected this purported isoniazid resistance in one of our clinical isolates.

In summary, we have shown isolation of *M. bovis* BCG strain is rare, and that the BCG vaccine is safe and well-tolerated in immunocompetent children. Only in the setting of profound immunocompromise do we see molecular and phenotypic rifampicin resistance emerging, most likely facilitated by a large bacillary burden. Because invasive disseminated BCG disease is rare, reporting and data sharing is important to allow assimilation of experience, particularly with respect to unusual cases of antibiotic resistance.

## Data availability

### Underlying data

European Nucleotide Archive: Clinical Mycobacterium bovis (BCG) isolates and antibiograms: a case series from children in Oxfordshire, 2007-2017. Accession number PRJNA548242; https://identifiers.org/ebi/ena.embl:PRJNA548242.
