# Supplementary material for: Case Report: Disseminated, rifampicin resistant Mycobacterium bovis (BCG) infection in an immunocompromised child
Source: Wellcome Open Res. 2020 Oct 15;5:242. [Version 1] doi: 10.12688/wellcomeopenres.16280.1 (PMC7682502; doi:10.12688/wellcomeopenres.16280.1)
Supplement: Peer review history

# Peer review: Reviewer response for version 1

**DOI:** 10.21956/wellcomeopenres.17888.r40940

**Published:** 2020-11-25

**Authors:** Mark P. Nicol

**Affiliations:** 1 Department of Microbiology, The University of Western Australia, Crawley, WA, Australia

This interesting and well-written case report (and series) complements our understanding of the risks associated with BCG vaccination.

 I have several minor queries/suggestions:

1. As noted by reviewer Ben Marais, it would be useful to know the specifics of INH susceptibility testing (concentration[s] of INH used), given the varying susceptibility reported, and the background low level resistance expected in this BCG strain.
2. Similarly, it would be fascinating to know whether there is evidence of a resistant sub-population within the BCG vaccine stock - i.e., whether we're vaccinating children with a heteroresistant strain.
3. I'm not sure that statements about the rarity of BCG-related adverse events is fully supported by the data, as there are only broad estimates of country-level denominators. Further, it is not completely clear whether **all** *M. tuberculosis* complex isolates over the period of study underwent WGS, or only a subset. 
4. Similarly, the statement that "it is the host response that is important in the development of disease rather than any specific attribute of the vaccine strain" does not appear to be fully substantiated by the evidence presented. All adverse events were associated with one strain, and there are no data given on the numbers of children vaccinated with different strains within the study region and period.
5. I am not sure that I fully agree with the statement that "in cases with local vaccine site collections or adenitis, it could be argued that laboratory diagnosis did not inform the management". Ruling out other causes of infection may have been clinically useful in the management of these children.

Are enough details provided of any physical examination and diagnostic tests, treatment given and outcomes?

Yes

Is the case presented with sufficient detail to be useful for other practitioners?

Yes

Is sufficient discussion included of the importance of the findings and their relevance to future understanding of disease processes, diagnosis or treatment?

Yes

Is the background of the case’s history and progression described in sufficient detail?

Yes

Reviewer Expertise:

clinical microbiology, tuberculosis, microbiome

I confirm that I have read this submission and believe that I have an appropriate level of expertise to confirm that it is of an acceptable scientific standard, however I have significant reservations, as outlined above.

# Peer review: Reviewer response for version 1

**DOI:** 10.21956/wellcomeopenres.17888.r41519

**Published:** 2020-11-20

**Authors:** Ben J. Marais

**Affiliations:** 1 Westmead Children's Hospital and the Marie Bashir Institute for Infectious Diseases and Biosecurity, University of Sydney, Sydney, NSW, Australia

Well written and interesting case series. Informed discussion and balanced conclusion.

 Minor comments for consideration:

1. Variability in DST and SNPs in Danish strain 1331 BCG (without nay treatment selection) is interesting. DST variability may be explained by inaccurate phenotypic results - it would be good to know the level of resistance detected in these instances. It is also relevant for INH, given that resistance was VERY rarely detected phenotypically, which is unexpected if tested at 0.1ug/ml. 
2. For SNP variability the question remains if this represents post administration diversity or diversity in the 'vaccine stock' itself. Have the authors tested 'vaccine stock' (straight from the vail) to assess possible SNP variability?
3. In the discussion it would be good to provide an estimated denominator for the statement that BCG adverse effects were VERY rare. Do we know how many BCGs (roughly) were administered to children in the study area over the study period?
4. Any suggestions on how the single case could/should have been dealt with differently to prevent the recurrent relapses observed? Should the initial treatment period have been extended in light of the immune abnormality and ineffectiveness of PZA?
5. Can we accept 'significant within host selective pressure' as a likely explanation in a child with significant immune compromise? It seems as if treatment induced selective pressure, differential drug penetration and selective sampling of bacterial sub-populations offer the most likely explanation in this instance.

Overall this is an informative and well-presented report that makes an important contribution to the existing literature. Consideration of minor comments above should help to refine it further.

Are enough details provided of any physical examination and diagnostic tests, treatment given and outcomes?

Yes

Is the case presented with sufficient detail to be useful for other practitioners?

Yes

Is sufficient discussion included of the importance of the findings and their relevance to future understanding of disease processes, diagnosis or treatment?

Yes

Is the background of the case’s history and progression described in sufficient detail?

Yes

Reviewer Expertise:

Tuberculosis

I confirm that I have read this submission and believe that I have an appropriate level of expertise to confirm that it is of an acceptable scientific standard.

# Peer review: Reviewer response for version 1

**DOI:** 10.21956/wellcomeopenres.17888.r40938

**Published:** 2020-11-04

**Authors:** Elizabeth Whitaker

**Affiliations:** 1 Department of Paediatrics, Imperial College London, London, UK

This is an interesting and well written case report highlighting the complexity of managing a rare case of disseminated BCG. It also showed the importance of phenotypic sensitivity testing to ensure the most appropriate anti-mycobacterial treatment course for this rare condition. It also explains why multiple drugs are included in regimens to treat patients such as these. The authors do not comment on toxicity/adverse drug reactions in this child. They also don't comment on drug levels - metabolism of rifampicin in children differs greatly to adults, and higher drug doses are required to achieve therapeutic drug levels - this child may have benefited from therapeutic drug monitoring - the authors could comment on its role in these complex cases. It may be that low levels of rifampicin contributed to the within-host selective pressure.

 As it is a case report, I would be interested to see more details on the clinical case, but understand why the authors have concentrated on the drug sensitivity element of the presentation - as this has relevance for the readers, in particular if they have a similar case in the future. I wonder if the team have considered the role of phage therapy for this child, as she seems to be still be on an antibiotic regimen 3 years later. 

 Reporting rare cases such as these has value as clinical decisions are made on expert opinion, rather than evidence based data. This case will ensure future similar cases are considered in the context of drug sensitivity/resistance. 

 It would be interesting for the authors to comment on screening for primary immune deficiencies and planned changes to BCG delivery in the UK. Of particular note, this child would not have been identified by TREC screening, so the case would not have been avoided.

Are enough details provided of any physical examination and diagnostic tests, treatment given and outcomes?

Partly

Is the case presented with sufficient detail to be useful for other practitioners?

Yes

Is sufficient discussion included of the importance of the findings and their relevance to future understanding of disease processes, diagnosis or treatment?

Yes

Is the background of the case’s history and progression described in sufficient detail?

Yes

Reviewer Expertise:

Tuberculosis, BCG, mycobacterial immunity immunodeficiency in children SARS-CoV-2

I confirm that I have read this submission and believe that I have an appropriate level of expertise to confirm that it is of an acceptable scientific standard.
